# Patient satisfaction and OHRQoL in single-piece implant full-mouth rehabilitation versus fixed bridges

**DOI:** 10.6026/973206300220401

**Published:** 2026-01-31

**Authors:** Sadaf Farheen, Kota Bharathi, Aditi Kanitkar, Deepika C.S, Remya Ravi, Padminii Ellapakurthi, Heena Dixit Tiwari

**Affiliations:** 1 Department of dental surgery, Mamata Dental College and Hospital, Khammam, Telangana, India; 2 Department of Periodontics, Mamata Institute of Dental Sciences, Bachupally, Hyderabad, Telangana, India; 3 Department of Prosthodontics, Bharati Vidyapeeth (Deemed to be University) Dental College and Hospital, Sangli, Maharashtra, India; 4 Department of Prosthodontics and Crown & Bridge, Azeezia College of Dental Sciences & Research, Kollam, Kerala, India; 5 Department of Prosthodontics, Azeezia College of Dental Sciences & Research, Kollam, Kerala, India; 6 Department of Oral and Maxillofacial Surgery, Karnavati School of Dentistry, Gandhinagar, Gujarat, India; 7 Blood Cell, Commissionerate of Health and Family Welfare, Government of Telangana, Hyderabad, India

**Keywords:** Dental Implants, oral health-related quality of life, fixed prosthesis, patient satisfaction, edentulous jaw rehabilitation

## Abstract

Complete edentulism rehabilitation remains challenging because conventional fixed bridges often fail to achieve optimal long-term patient satisfaction and oral health-related quality of life. Therefore, it is of interest to compare patient satisfaction and oral health-related quality of life (OHRQoL) between single-piece implant-supported full-mouth rehabilitation and conventional fixed bridges in completely edentulous patients. Results demonstrated significantly higher OHRQoL and satisfaction scores among implant-supported cases, emphasizing improved functional stability, aesthetics and psychological well-being. Thus, single-piece implants achieved superior patient-reported outcomes and greater rehabilitation predictability.

## Background:

Complete edentulism restoration improved with implant-supported prostheses. Conventional fixed bridges show biomechanical and hygiene limitations. Implantology enhances oral function, aesthetics and prosthetic stability [1]. Single- piece implants use monobloc design without abutment junction [2]. This reduces micro-gap complications and peri-implantitis risk. Immediate loading shortens treatment time and reduces patient distress [3]. Conventional fixed bridges depend on teeth and cause biological stress [4]. They may lead to bone loss and occlusal imbalance. Patient-reported outcome measures assess rehabilitation experience effectively [5]. Implant-supported prostheses improve speech, chewing, aesthetics and self-esteem [6]. Few studies compare single-piece implant full-mouth rehabilitation outcomes [7]. Therefore, it is of interest to compare satisfaction and OHRQoL between treatments.

## Materials and Methods:

## Study design and population:

A prospective comparative clinical study was conducted on 40 edentulous patients aged 45-70 years.

## Participants were divided into two equal groups:

[1] Group I: Single-piece implant-supported full-mouth rehabilitation (n=20)

[2] Group II: Conventional fixed bridges supported by natural abutments (n=20)

## Inclusion and exclusion criteria:

Patients with systemic health permitting implant surgery (ASA I-II), adequate bone support (D2-D3 bone) and absence of severe parafunctional habits were included. Individuals with uncontrolled diabetes, smoking, or recent bisphosphonate therapy were excluded.

## Intervention protocol:

[1] In Group I, single-piece corticobasal implants (immediate loading, basal cortical screw type) were placed following flapless protocols. Definitive fixed hybrid prostheses were delivered within 72 hours.

[2] In Group II, full-mouth rehabilitations were performed using conventional porcelain-fused-to-metal fixed bridges on prepared natural teeth.

## Assessment tools:

[1] Oral Health Impact Profile (OHIP-14) was used to evaluate OHRQoL pre- and post-rehabilitation.

[2] Visual Analog Scale (VAS) was used for overall satisfaction (0 = poor, 10 = excellent).

[3] Follow-up periods: Baseline, 3 months and 12 months post-prosthesis delivery.

## Statistical analysis:

Data were analyzed using SPSS v25. Paired t-test and ANOVA assessed intragroup and intergroup differences. p < 0.05 was considered statistically significant.

## Results:

Patients in the single-piece implant group demonstrated significantly lower OHIP-14 scores across all domains, reflecting superior comfort, function and psychosocial well-being compared to conventional fixed bridge patients. Functional and psychological domains showed the most notable improvement Table 1. Single-piece implant patients showed significantly greater satisfaction in mastication, speech and esthetic outcomes. Hygiene maintenance scores were comparable. High overall satisfaction indicates improved patient adaptation and psychological acceptance of the implant modality Table 2.

## Discussion:

This study assessed satisfaction and OHRQoL after two rehabilitation methods. Single-piece implant restorations improved function, aesthetics and psychology. Implant rehabilitations restore chewing and speech near natural dentition [8]. Monobloc design reduces micro-movement and bacterial leakage [9]. This improves peri-implant tissue stability and clinical outcomes. Manfredini *et al.* reported reduced OHIP-14 and better VAS scores [9]. Awadalkreem *et al.* showed 100% survival with corticobasal implants [10]. Conventional bridges may cause abutment fatigue and pulpal damage [11]. Higher OHIP scores confirm limitations of tooth-supported prostheses. Satisfaction depends on invasiveness, cost, speech and maintenance [12]. Borges *et al.* found implant prostheses improve comfort and mastication [13]. Implants restore self-image and social confidence effectively. Srinivasan *et al.* confirmed better psychosocial outcomes with implants [14]. Single-piece implants transmit load directly to cortical bone. This minimizes crestal bone loss and improves predictability [15]. Tooth-supported prostheses may increase ridge resorption and discomfort. Hygiene challenges exist in both rehabilitation methods [16]. PROMs now complement survival-based implant success criteria [17]. Recent evidence supports PROMs with bone loss assessment [18]. This study updates knowledge by providing direct comparative evidence on single-piece implant full-mouth rehabilitation using standardized OHRQoL and satisfaction measures. It strengthens patient-centered decision-making in complete edentulism treatment.

## Limitations:

This study was limited by its small sample size and short follow-up period. Longitudinal multicentric trials are warranted to substantiate the long-term psychosocial and functional impacts of single-piece implant systems.

## Clinical implications:

Single-piece implants are a viable and efficient full-mouth rehabilitation modality, offering superior satisfaction, functional stability and OHRQoL. Clinicians should consider patient expectations, cost factors and maintenance compliance before treatment selection [19, 20].

## Conclusion:

Single-piece implant-supported full-mouth rehabilitation provides superior patient satisfaction and OHRQoL outcomes compared to conventional fixed bridges. These findings advocate single-piece implant protocols as predictable, minimally invasive and patient- centered approaches in comprehensive oral rehabilitation.

## Figures and Tables

**Table 1:** Mean OHIP-14 domain scores between groups

**Domain (OHIP-14)**	**Group I (Implant) Mean ± SD**	**Group II (Bridge) Mean ± SD**	***p-value***
Functional Limitation	2.1 ± 0.6	4.8 ± 1.1	<0.001
Physical Pain	1.8 ± 0.5	3.9 ± 0.9	<0.001
Psychological Discomfort	1.6 ± 0.7	3.5 ± 1.0	<0.001
Physical Disability	2.0 ± 0.8	4.3 ± 1.2	<0.001
Psychological Disability	1.5 ± 0.5	3.7 ± 0.9	<0.001
Social Disability	1.3 ± 0.6	3.2 ± 1.0	<0.001
Handicap	1.4 ± 0.5	3.6 ± 1.1	<0.001

**Table 2:** Mean VAS Scores for Patient Satisfaction

**Parameter**	**Group I (Implant) Mean ± SD**	**Group II (Bridge) Mean ± SD**	***p-value***
Mastication	9.3 ± 0.5	7.2 ± 0.9	<0.001
Speech	9.0 ± 0.7	7.5 ± 0.8	<0.01
Esthetics	9.1 ± 0.6	8.0 ± 0.7	<0.05
Hygiene Maintenance	8.5 ± 0.8	7.8 ± 0.6	0.06 (NS)
Overall Satisfaction	9.2 ± 0.5	7.6 ± 0.9	<0.001
